# Contrasting selection pressure on body and weapon size in a polygynous megaherbivore

**DOI:** 10.1098/rsbl.2021.0368

**Published:** 2021-10-06

**Authors:** Graeme Shannon, Phoebe Sadler, Joanna Smith, Eleanor Roylance-Casson, Line S. Cordes

**Affiliations:** ^1^ School of Natural Sciences, Bangor University, Bangor, Gwynedd LL57 2DG, UK; ^2^ School of Ocean Sciences, Bangor University, Menai Bridge, Anglesey LL59 5AB, UK

**Keywords:** sexual size dimorphism, common hippo, *Hippopotamus amphibius*, evolution, male–male competition

## Abstract

Sexual size dimorphism (SSD) is a common morphological trait in ungulates, with polygyny considered the leading driver of larger male body mass and weapon size. However, not all polygynous species exhibit SSD, while molecular evidence has revealed a more complex relationship between paternity and mating system than originally predicted. SSD is, therefore, likely to be shaped by a range of social, ecological and physiological factors. We present the first definitive analysis of SSD in the common hippopotamus (*Hippopotamus amphibius*) using a unique morphological dataset collected from 2994 aged individuals. The results confirm that hippos exhibit SSD, but the mean body mass differed by only 5% between the sexes, which is rather limited compared with many other polygynous ungulates. However, jaw and canine mass are significantly greater in males than females (44% and 81% heavier, respectively), highlighting the considerable selection pressure for acquiring larger weapons. A predominantly aquatic lifestyle coupled with the physiological limitations of their foregut fermenting morphology likely restricts body size differences between the sexes. Indeed, hippos appear to be a rare example among ungulates whereby sexual selection favours increased weapon size over body mass, underlining the important role that species-specific ecology and physiology have in shaping SSD.

## 1. Introduction

Sexual size dimorphism (SSD) is a common morphological trait whereby male and female animals exhibit significant differences in body size [1]. In most species, females are the larger sex because body size is positively correlated with fecundity. However, in mammals, the opposite relationship predominates [2], and comparative studies suggest that these morphological differences are mainly driven by divergence in reproductive investment [3,4]. Females spend considerable energy and time on gestation and weaning, while males rarely have much parental input beyond copulation. As a result, males can focus their efforts on maximizing the number of mating opportunities that they can secure. This often leads to pronounced male–male competition, which has been proposed as the main selective force in the emergence of SSD among polygynous mammals [5–8].

 SSD is particularly common in ungulates with males being at least 10% larger in two-thirds of species (unpublished data [9]). Weapon sizes are also generally more pronounced in males than females, especially among polygynous species [10]. These findings provide further support for the fitness benefits of being larger and better equipped to win contests and outcompete other males [11]. The expansion of open grassland habitats during the Miocene is believed to be the key mechanism underpinning the evolution of divergent body sizes in ungulates, as this enabled animals to aggregate in larger groups providing the opportunity for the most dominant males to secure mating opportunities with multiple females [8,12,13]. Indeed, the trend for SSD to predominate in environments where females aggregate is observed across ungulate species [8].

It is important to highlight that not all polygynous ungulate species exhibit SSD, including for example, equids [14] and peccaries [15], while molecular analysis has begun to reveal that the largest and strongest males do not always outcompete smaller rivals and monopolize access to oestrus females [16]. In fact, when paternity is accounted for, the relationship between sexual dimorphism and polygyny is often less pronounced than originally predicted [17,18]. These findings suggest that the specific nature of contests between males, the extent of sexual segregation, physiological constraints and environmental conditions are also likely to play a key role in driving SSD in ungulates [19]. Finally, it is also important to consider the competitive selection pressures that act on females to maximize body size, which can reduce or even reverse SSD [20,21].

In this study, we explore the extent of sexual dimorphism in body size and weaponry for one of the largest and yet one of the least studied ungulate species, the common hippopotamus (*Hippopotamus amphibius*). The hippo is classified as a megaherbivore (greater than 1000 kg in weight); however, there are a wide range of body size estimates for males and females with conflicting evidence regarding whether hippos actually exhibit SSD, and if so, to what extent [22–26]. They have been classified either as highly dimorphic with males being up to 40% larger than females [24,26] or moderately dimorphic in body mass (males approx. 10% heavier) with negligible differences in length and height of the sexes [22,23]. However, small sample sizes, unknown ages and a lack of formal statistical analysis have limited the precision of these estimates.

Hippos are challenging animals to study, they spend the majority of their time in water only emerging at night to feed; individual identification is difficult, and they are notoriously aggressive [23,24]. Nevertheless, it is well established that hippos exhibit a polygynous breeding system whereby dominant males aggressively defend water-based territories (ranging from 50 to 500 m in length) while monopolizing access to multiple females within these comparatively small stretches of water [23,27]. We, recently, acquired an unrivalled dataset on hippo age and sex-specific morphology, which enabled us to definitively explore the magnitude of SSD within this species for the first time. We predicted that the high level of male–male competition is likely to generate strong selection pressure for SSD [28]. As such, males will have significantly larger body size and weapons (tusks or lower canines) than adult females. Furthermore, we expected these morphological differences to be particularly pronounced given Rensch's rule, which states that sexual dimorphism increases with body mass in taxa where males are the larger sex [2].

## 2. Material and methods

### (a) Data collection

The data were collected in Queen Elizabeth National Park (QENP), Uganda from 1961 to 1966 by Prof. Richard Laws (Nuffield Unit of Tropical Animal Ecology) and his research team. QENP is 1978 km^2^ in extent and is located in the southwest of Uganda. In the early 1960s, QENP was home to a population of approximately 15 000 hippos [29]. Detailed morphological measurements were collected from 2994 hippos culled in and around Lake Edward as a management intervention to reduce overgrazing.

The animals were weighed in pieces on a spring balance. Body length measurements were read from a steel tape and mandible measurements were made with calipers. [Lower] jaw weights were taken with a spring balance to the nearest 50 g; canine and incisor weights to the nearest 25 g [30, p. 20].

All of the hippos were sexed and subsequently aged using jaw characteristics, tooth replacement and tooth wear before being assigned to one of 15 rigorously defined age categories from 1 to 35 years (see [30] for full methodology). The mean age of each category was used in our analysis providing an unparalleled level of morphological data across the lifespan of this enigmatic species. The canine mass data were extracted directly from figs 11 and 12 in [30] using WebPlotDigitizer (https://automeris.io/WebPlotDigitizer/). All measurements were converted to SI units.

### (b) Data analysis

Two sets of analyses were conducted. Firstly, we explored the development of SSD with age using three metrics: body mass (kg), body length (cm) and shoulder height (cm). Secondly, we explored SSD in weapon size using two metrics: lower jaw mass (kg) and combined canine mass (kg), as a function of body size. Body length was selected as the explanatory variable as these data were more abundant compared to body mass. However, body size measurements were not available for the canine mass data and, therefore, age was used as a proxy of body size for this analysis.

Generalized additive models (GAMs) were applied to all model sets using the mgcv package [31] in R [32], as preliminary analyses using linear regression of logged variables and GLMs revealed patterns in the residuals during the model validation process. We constructed single parameter models including only age or body length, two-parameter models including the additive effect of sex, and interactions between age or body length and sex. To model growth relationships that were biologically realistic, we only constructed models with three or four knots (*k* = endpoints where polynomials meet), thereby controlling the dimensionality and hence reducing the complexity of the fitted smooth function. Model selection was conducted using AICc selecting the top model with the lowest AICc score, which was then used for model validation. The simplest model was chosen if the top models were within two AICc scores of each other. A Welch two-sample *t*-test was used to compare metrics between male and female adult hippos. From the GAM results, adulthood was assumed to commence around 10 years of age (equivalent to a mean body length of 318 cm). Data are available in the Dryad Digital Repository [33].

## 3. Results

**Figure 1.**
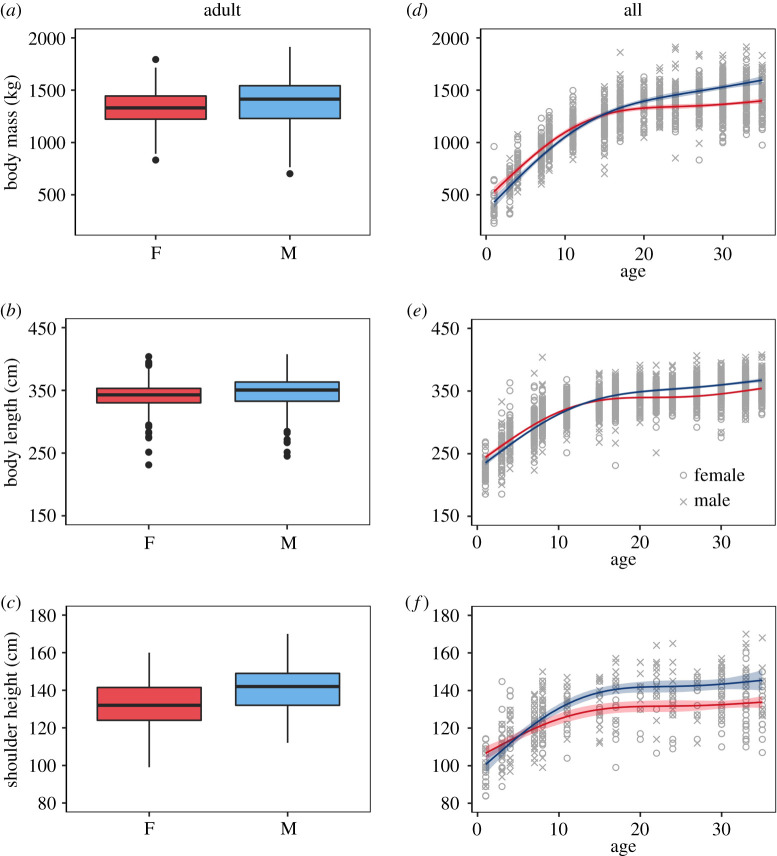
Box and whisker plots illustrating the sex-specific differences in body size (mass, length and height) of adult hippos (*a*–*c*), and the age-specific development of body size in female (red) and male (blue) hippos (*d*–*f*). Adults were designated as individuals greater than or equal to 10 years of age.

**Figure 2.**
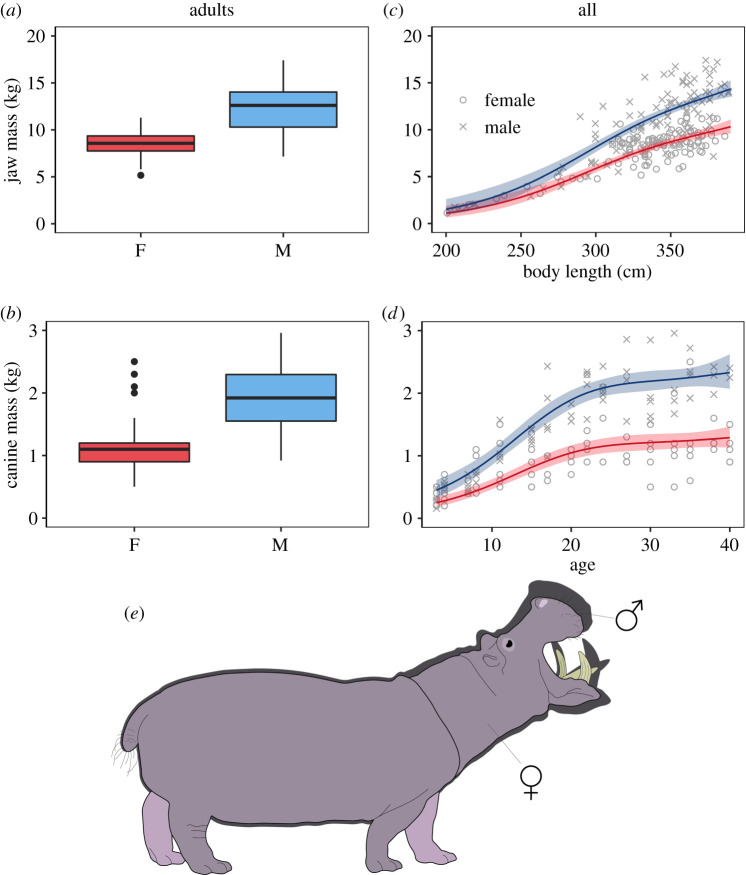
Box and whisker plots illustrating the sex-specific differences in weapon size (jaw and canine mass) of adult hippos (*a*,*b*), and the age-specific development of weapon size in female (red) and male (blue) hippos (*c*,*d*). Adults were designated as individuals greater than or equal to 10 years of age or greater than or equal to 318 cm long. A visual representation showing the extent of SSD in an average adult male and female hippo (*e*). All artwork was created by L.S.C.

Adult male hippos were significantly heavier (5%), longer (2%) and taller (7%) than females (figure 1*a–c*; electronic supplementary material, table S1). However, the relative difference in jaw and canine mass was much more pronounced with the jaws of males being 44% heavier and canines 81% heavier compared to females (figure 2*a,b*). There was a clear top model for all three body size model sets, where the relationship between the body size metrics and age was best described by a GAM with four knots including sex as an interaction (electronic supplementary material, table S2). The increase in body mass and length was similar for males and females until the age of approximately 17 where males continued to grow but females levelled off (figure 1*d*,*e*). Both sexes reached maximum height around the age of approximately 15, while younger males exhibited faster growth (figure 1*f*). Although males were 7% taller, girth measurements revealed that this linear metric of body size did not translate into a larger relative increase in body mass—as might be expected—because males were 5% leaner than females (see electronic supplementary material, figure S1).

Models including an interaction between length (or age) and sex were technically the top models for our metrics of weapon size (electronic supplementary material, table S3). However, the delta AICc between the top models and the second-best models were only 0.3 and 2 for jaw and canine mass, respectively. Therefore, the second-best models were selected as these were simpler, including sex as an additive effect and a smoother with either three or four knots. Jaw mass increased faster in young males compared to females (figure 2*c*) while the rate of growth started slowing down for both sexes around a length of approximately 320 cm. Canines grew much faster in younger males compared to females, with growth levelling off around the age of 20 (figure 2*d*).

## 4. Discussion

The analysis of this detailed dataset on hippo age-specific morphology revealed that adult males are on average heavier, longer and taller than adult females—providing the first definitive evidence that hippos exhibit differences in body size between the sexes. However, these differences are relatively small compared with many other polygynous ungulates [3,8,9]. It seems unlikely that a 60 kg difference in the mean body mass of an adult male hippo (which can exceed 1500 kg) is biologically significant when competing for access to females. Indeed, compared with the African elephant, a megaherbivore that also exhibits intense male–male competition, the extent of body size dimorphism in hippos is negligible. A fully grown male elephant can be twice the weight of an adult female with tusks that weigh five to seven times as much [34]. Our results also revealed that among young hippos, the females were slightly larger than males, which is the opposite of what is observed in most sexually dimorphic mammals [35].

 Intriguingly, weapon size metrics demonstrated a much greater divergence between male and female hippos. Male jaw masses were on average 44% heavier and canines were nearly twice the weight of those found in adult females, with males investing considerably in weapon growth during early adulthood (10–20 years). In fact, the larger male head likely accounts for a significant proportion of the disparity in body mass between the sexes. These results suggest that there is much greater selection pressure for males to acquire larger weapons to dominate and outcompete rivals, rather than investing in body size (figure 2*e*). This is further supported by the fact that the post-canine teeth—predominantly used for feeding—did not differ in size between the sexes [30]. Selection for weapons over body size is comparatively rare in male ungulates, which generally exhibit a positive allometric relationship between these characteristics [10,36]. In bovids, the rate of growth in weapons was actually found to decrease relative to body size, as horn size was either constrained or no longer the target of sexual selection [36].

Aggressive displays and interactions between rival hippos generally take place in water [23,27] where body size may be less influential in male–male competition than on land, particularly as water can negate the weight advantage and obscure the visual assessment of body size. Indeed, pinnipeds that breed in water exhibit very limited body size dimorphism compared with species that breed on land, while female baleen whales are generally larger than males [37]. Male hippos commonly signal dominance by yawning and displaying the gape of their jaw and the size of their tusks [23]. Therefore, selection pressure for larger weapons appears to outstrip the need for increased body size. Evidence for greater selection pressure on canine size compared with body size has also been found among a number of primates [38] and carnivore species [39], where intense competition for access to females is predominantly mediated by the display (and occasional use) of these weapons.

The digestive physiology of hippos may also constrain body size dimorphism, as they are the largest species employing foregut fermentation to process their food [23]. Whereas rhinos and elephants both use hind-gut fermentation, which enables much greater throughput of abundant lower quality forage despite some decreased digestive efficiency [40]. Hippos are very well adapted to the shorter feeding bouts and longer forage retention times required by foregut fermentation, particularly as they exhibit significantly lower energy expenditure compared with other megaherbivores due to the buoyancy and reduced thermal stress afforded by living in water [23]. However, the limits on the rate of ingestion have been shown to place much greater constraints on maximum attainable body size compared with hind-gut fermenters [40,41]. Interestingly, the greater one-horned rhinoceros—the second largest rhino species—exhibits a similar pattern in body and weapon size to the hippo. They are largely monomorphic due to dietary constraints, but the males have significantly larger incisors that they use for fighting (slashing and gouging), and much more developed neck and shoulder muscles [42].

Finally, selection pressure could also be acting on female hippos to maximize their body size in order to protect their young and compete successfully for access to water and forage during periods of resource scarcity—the big mother hypothesis [20]. This selection pressure can potentially reduce any differences that may accrue from intra-sexual selection, or even result in females with larger morphological traits, such as in the Korean water deer [43].

Our study uses a unique dataset on hippo morphology to demonstrate convincingly that body size dimorphism is comparatively limited for this large-bodied and highly polygynous ungulate. Yet, there appears to be considerable intra-sexual selection for larger weapon size among males, which is driven by intense competition for mating opportunities. The hippo provides a fascinating example of a species where ecology and physiology likely constrain the extent of body size dimorphism between the sexes, but where weapon size plays a primary role in establishing dominance and securing mating rights. These findings further underline how SSD is a complex trait that is shaped and constrained by more than just the reproductive strategies of the sexes.

## Data accessibility

Data used in this study are published in the Dryad Digital Repository: https://doi.org/10.5061/dryad.ttdz08kzw.

## References

[1] Mori E, Mazza G, Lovari S. 2017 Sexual dimorphism. In Encyclopedia of animal cognition and behavior (eds J Vonk, T Shakelford), pp. 1-7. Cham, Switzerland: Springer International Publishing.

[2] Abouheif E, Fairbairn DJ. 1997 A comparative analysis of allometry for sexual size dimorphism: assessing Rensch's rule. Am. Nat. **149**, 540-562. (10.1086/286004)

[3] Lindenfors P, Gittleman JL, Jones KE. 2007 Sexual size dimorphism in mammals. In Sex, size and gender roles: evolutionary studies of sexual size dimorphism (eds DJ Fairbairn, WU Blanckenhorn, T Székely), pp. 16-26. Oxford, UK: Oxford University Press.

[4] Soulsbury CD, Kervinen M, Lebigre C. 2014 Sexual size dimorphism and the strength of sexual selection in mammals and birds. Evol. Ecol. Res. **16**, 63-76.

[5] Lindenfors P, Tullberg BS, Biuw M. 2002 Phylogenetic analyses of sexual selection and sexual size dimorphism in pinnipeds. Behav. Ecol. Sociobiol. **52**, 188-193. (10.1007/s00265-002-0507-x)

[6] Weckerly FW. 1998 Sexual-size dimorphism: influence of mass and mating systems in the most dimorphic mammals. J. Mammal. **79**, 33-52. (10.2307/1382840)

[7] Loison A, Gaillard J-M, Pélabon C, Yoccoz NG. 1999 What factors shape sexual size dimorphism in ungulates? Evol. Ecol. Res. **1**, 611-633.

[8] Pérez-Barbería FJ, Gordon IJ, Pagel M. 2002 The origins of sexual dimorphism in body size in ungulates. Evolution (N Y) **56**, 1276-1285. (10.1111/j.0014-3820.2002.tb01438.x) 12144026

[9] Roylance-Casson E. 2021 Rensch's rule and the drivers of sexual dimorphism in ungulates. MSc thesis. Bangor University, Bangor, Gwynedd, UK. See https://research.bangor.ac.uk/portal/files/36593392/Roylance_Casson_thesis_20212021RoylanceCassonEKMscRes_1.pdf.

[10] Bro-Jørgensen J. 2007 The intensity of sexual selection predicts weapon size in male bovids. Evolution (N Y) **61**, 1316-1326. (10.1111/j.1558-5646.2007.00111.x) 17542842

[11] Rico-Guevara A, Hurme KJ. 2019 Intrasexually selected weapons. Biol. Rev. **94**, 60-101. (10.1111/brv.12436) 29924496

[12] Jarman PJ. 1974 The social organisation of antelope in relation to their ecology. Behaviour **48**, 215-267. (10.1163/156853974x00345)

[13] Szemán K, Liker A, Székely T. 2021 Social organization in ungulates: revisiting Jarman's hypotheses. J. Evol. Biol. **34**, 604-613. (10.1111/jeb.13782) 33706412

[14] Houpt KA, Keiper R. 1982 The position of the stallion in the equine dominance hierarchy of feral and domestic ponies. J. Anim. Sci. **54**, 945-950. (10.2527/jas1982.545945x)

[15] Leite DA, Keuroghlian A, Rufo DA, Miyaki CY, Biondo C. 2018 Genetic evidence of promiscuity in a mammal without apparent sexual dimorphism, the white-lipped peccary (*Tayassu pecari*). Mamm. Biol. **92**, 111-114. (10.1016/j.mambio.2018.05.005)

[16] Isvaran K, Sankaran S. 2017 Do extra-group fertilizations increase the potential for sexual selection in male mammals? Biol. Lett. **13**, 20170313. (10.1098/rsbl.2017.0313) 29070588 PMC5665768

[17] Cassini MH. 2020 A mixed model of the evolution of polygyny and sexual size dimorphism in mammals. Mamm. Rev. **50**, 112-120. (10.1111/mam.12171)

[18] Isaac JL. 2005 Potential causes and life-history consequences of sexual size dimorphism in mammals. Mamm. Rev. **35**, 101-115. (10.1111/j.1365-2907.2005.00045.x)

[19] Bowyer RT, McCullough DR, Rachlow JL, Ciuti S, Whiting JC. 2020 Evolution of ungulate mating systems: integrating social and environmental factors. Ecol. Evol. **10**, 5160-5178. (10.1002/ece3.6246) 32551090 PMC7297761

[20] Ralls K. 1976 Mammals in which females are larger than males. Q. Rev. Biol. **51**, 245-276. (10.1086/409310) 785524

[21] Stockley P, Bro-Jørgensen J. 2011 Female competition and its evolutionary consequences in mammals. Biol. Rev. **86**, 341-366. (10.1111/j.1469-185X.2010.00149.x) 20636474

[22] Owen-Smith RN. 1988 Megaherbivores: the influence of very large body size on ecology. Cambridge, UK: Cambridge University Press.

[23] Eltringham SK. 1999 The hippos: natural history and conservation. Princeton, NJ: Princeton University Press.

[24] Estes RD. 2012 The behavior guide to African mammals: including hoofed mammals, carnivores, primates. Berkeley, CA: University of California Press.

[25] Weston EM. 2003 Evolution of ontogeny in the hippopotamus skull: using allometry to dissect developmental change. Biol. J. Linn. Soc. **80**, 625-638. (10.1111/j.1095-8312.2003.00263.x)

[26] Pluháček J, Steck BL. 2015 Different sex allocations in two related species: the case of the extant hippopotamus. Ethology **121**, 462-471. (10.1111/eth.12357)

[27] Stears K, Nuñez TA, Muse EA, Mutayoba BM, McCauley DJ. 2019 Spatial ecology of male hippopotamus in a changing watershed. Sci. Rep. **9**, 1-13. (10.1038/s41598-019-51845-y) 31659224 PMC6817855

[28] Wade MJ, Shulter SM. 2004 Sexual selection: harem size and the variance in male reproductive success. Am. Nat. **164**, E83-E89. (10.1086/424531) 15459886

[29] Laws R, Blix AS, Parker I, Parker C. 2017 Large animals and wide horizons. Cambridge, UK: Janus Publishing Company Ltd.

[30] Laws RM. 1968 Dentition and ageing of the hippopotamus. Afr. J. Ecol. **6**, 19-52. (10.1111/j.1365-2028.1968.tb00899.x)

[31] Wood S, Wood MS. 2015 Package ‘mgcv’. *R Package* version 1, 29.

[32] R Core Team. 2018 R: a language and environment for statistical computing. Vienna, Austria: R Foundation for Statistical Computing. See http://www.R-project.org/.

[33] Shannon G, Sadler P, Smith J, Roylance-Casson E, Cordes LS. 2021Data from: Contrasting selection pressure on body and weapon size in a polygynous megaherbivore. *Dryad Digital Repository*. (10.5061/dryad.ttdz08kzw) PMC8492169 34610251

[34] Poole JH. 1994 Sex differences in the behaviour of African elephants. In The differences between the sexes (eds R Short, E Balaban), pp. 331-346. Cambridge, UK: Cambridge University Press.

[35] Clutton-Brock T. 2016 Mammal societies. Chichester, UK: John Wiley & Sons.

[36] Tidière M, Lemaître JF, Pélabon C, Gimenez O, Gaillard JM. 2017 Evolutionary allometry reveals a shift in selection pressure on male horn size. J. Evol. Biol. **30**, 1826-1835. (10.1111/jeb.13142) 28703357

[37] Mesnick S, Ralls K. 2018 Sexual dimorphism. In Encyclopedia of marine mammals (eds BG Würsig, JGM Thewissen, KM Kovacs), pp. 848-853. Amsterdam, The Netherlands: Elsevier.

[38] Thorén S, Lindenfors P, Kappeler PM. 2006 Phylogenetic analyses of dimorphism in primates: evidence for stronger selection on canine size than on body size. Am. J. Phys. Anthropol. **130**, 50-59. (10.1002/ajpa.20321) 16345072

[39] Gittleman JL, Van Valkenburgh B. 1997 Sexual dimorphism in the canines and skulls of carnivores: effects of size, phylogeny, and behavioural ecology. J. Zool. **242**, 97-117. (10.1111/j.1469-7998.1997.tb02932.x)

[40] Clauss M, Frey R, Kiefer B, Lechner-Doll M, Loehlein W, Polster C, Rössner GE, Streich WJ. 2003 The maximum attainable body size of herbivorous mammals: morphophysiological constraints on foregut, and adaptations of hindgut fermenters. Oecologia **136**, 14-27. (10.1007/s00442-003-1254-z) 12712314

[41] Clauss M, Jürgen Streich W, Schwarm A, Ortmann S, Hummel J. 2007 The relationship of food intake and ingesta passage predicts feeding ecology in two different megaherbivore groups. Oikos **116**, 209-216. (10.1111/j.2006.0030-1299.15461.x)

[42] Dinerstein E. 1991 Sexual dimorphism in the greater one-horned rhinoceros (*Rhinoceros unicornis*). J. Mammal. **72**, 450-457. (10.2307/1382127)

[43] Kim YK, Koyabu D, Lee H, Kimura J. 2013 Sexual dimorphism of craniomandibular size in the korean water deer, *Hydropotes inermis argyropus*. J. Vet. Med. Sci. **75**, 1153-1159. (10.1292/jvms.13-0125) 23615122

